# Methotrexate inhibits BMP4 and abrogates the hypertrophic chondrocyte phenotype of synovial fibroblasts in juvenile idiopathic arthritis

**DOI:** 10.1186/s12969-023-00940-6

**Published:** 2024-01-02

**Authors:** Megan M. Simonds, Samuel T. Freer, Anne Marie C. Brescia

**Affiliations:** 1 Nemours Biomedical Research, 1600 Rockland Rd, Wilmington, DE 19803 USA; 2 Nemours Children’s Health, Delaware, 1600 Rockland Rd, Wilmington, DE 19803 USA

**Keywords:** Juvenile idiopathic arthritis, Methotrexate, Bone morphogenetic protein 4, Synoviocytes, Chondrocytes

## Abstract

**Background:**

Juvenile Idiopathic Arthritis (JIA) induces growth disturbances in affected joints. Fibroblast-like synoviocytes (FLS) play a crucial role in JIA pathogenesis. FLS overexpress bone morphogenetic protein 4 (BMP4) and have a chondrocyte-like phenotype. FLS contribute directly to joint growth disturbances through endochondral bone formation. We investigated the ability of methotrexate to inhibit BMP4 expression and alter the hypertrophic chondrocyte-like phenotype of JIA FLS.

**Methods:**

We selected primary cells from three subjects with persistent oligoarticular JIA, three subjects who eventually extended to a polyarticular disease course, which we termed extended-to-be (ETB), and three subjects who had polyarticular arthritis at time of diagnosis. We treated cells with methotrexate and two BMP4 inhibitors: noggin and chordin. We measured protein concentration from three chondrocyte cell markers: collagen II, aggrecan, and collagen X as well as BMP4.

**Results:**

ColX, marker of chondrocyte hypertrophy, was significantly increased in polyarticular FLS when compared to both persistent FLS and ETB FLS, making polyarticular FLS the most like hypertrophic chondrocytes. Methotrexate caused significant decreases in BMP4 and ColX expression in persistent, ETB, and polyarticular FLS when compared to respective untreated cells. Ligand-binding BMP4 antagonists, noggin and chordin, caused significant decreases in ColX expression in FLS from all three disease courses and significant increases in collagen II protein, an early chondrocyte marker, when compared to respective untreated cells.

**Conclusions:**

Methotrexate, the first-line therapy in the treatment of JIA, mimics BMP4 antagonists by effectively lowering BMP4 and ColX expression in FLS. Inhibiting FLS from undergoing hypertrophy could prevent these cells from contributing to joint growth disturbances via endochondral bone formation.

**Supplementary Information:**

The online version contains supplementary material available at 10.1186/s12969-023-00940-6.

## Background

**Fig. 1:**
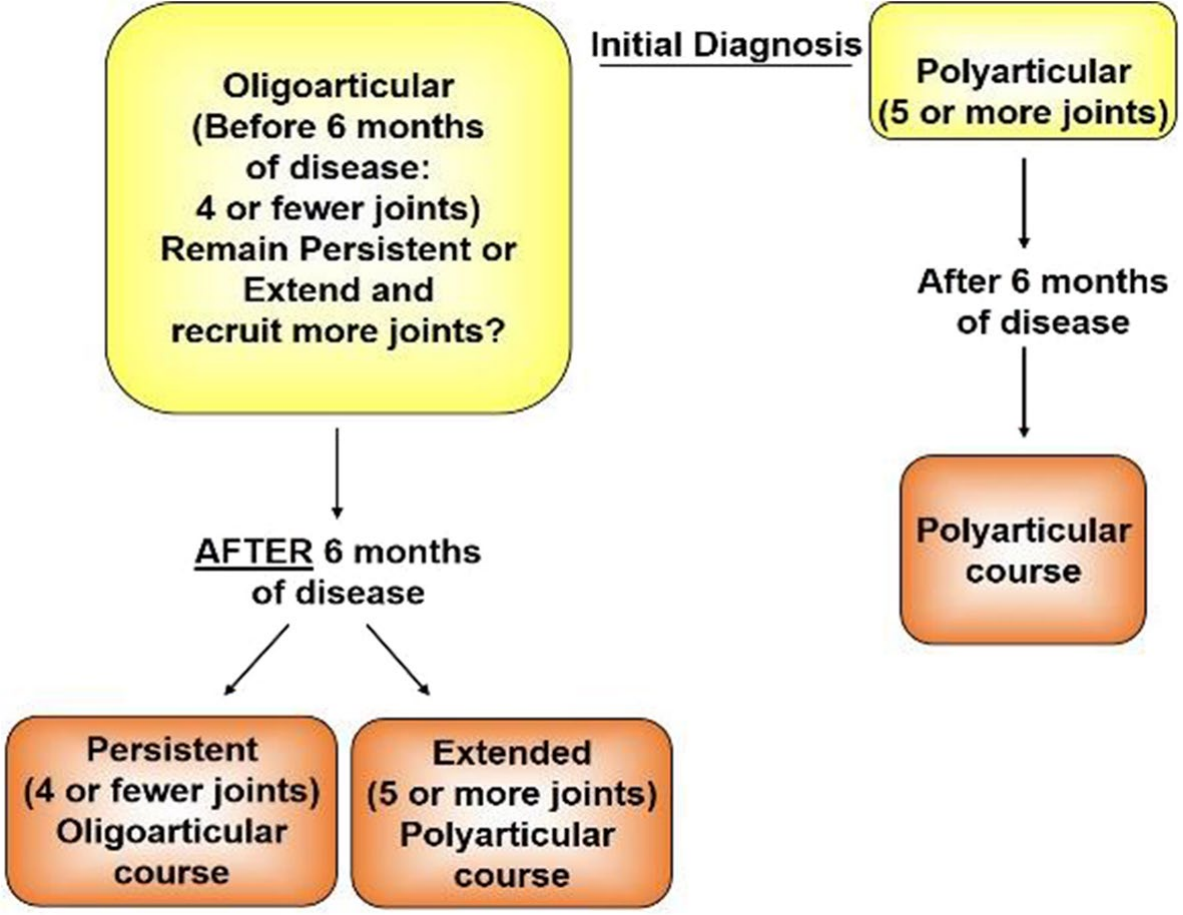
Schematic of evolution of JIA subtypes after 6 months of disease. It is unknown which patients will extend to polyarticular course prior to extension

Juvenile idiopathic arthritis (JIA) is the most common rheumatic disease of childhood and carries a risk of permanent joint damage and disability. The current classification scheme for JIA includes seven subtypes of disease based on onset [1, 2]. Although there can be ocular complications, the oligoarticular subtype has the most benign course of joint involvement, yet musculoskeletal disability results when joint involvement evolves from an oligoarticular to a polyarticular course, termed extended oligoarticular disease. Persistent oligoarthritis (persistent) affects up to 4 joints throughout the disease course, whereas extended oligoarthritis affects a cumulative total of 5 joints, or more after the first 6 months of disease [1] (Fig. 1). Between 21 and 50% of patients with oligoarticular onset show extension to a polyarticular course (polyarticular), of whom only 13–23% achieve disease-free remission [3–8]. Compared to patients with persistent oligoarticular course or polyarticular onset, patients with extended oligoarthritis have the lowest physical and mental scores in health-related quality-of-life assessments [9], with 38% ultimately requiring joint replacement, compared with only 13% of those with a persistent oligoarticular course [10].

In JIA, one factor of disease pathology is joint growth disturbances that can lead to leg-length discrepancies and disability [11, 12]. To date, targeted intraarticular corticosteroid injections have been effective at reducing inflammation in affected joints and may prevent leg length discrepancies [13]; however, while infrequent, the side effects to long-term steroid use can include systemic effects of steroids, infectious arthritis and cartilage damage [14].

In this study, we investigated the chondrocyte-like phenotype of fibroblast-like synoviocytes (FLS) and suggest that these cells may contribute directly to the bony overgrowth that occurs during disease progression. We have previously shown that FLS overexpress bone morphogenetic protein 4 (BMP4) and differentiate as hypertrophic chondrocytes, creating an environment favorable for endochondral bone formation (EBF) [15, 16]. We examined changes to the chondrocyte-like phenotype of FLS from three JIA subtypes: persistent, FLS isolated from synovial fluid of patients prior to extension to a polyarticular course or what we termed extended-to-be (ETB), and polyarticular in response to BMP4 inhibitors and methotrexate, the mainstay of JIA treatment. During EBF, BMP4 causes chondrocytes to hypertrophy [17]. These hypertrophic cells undergo apoptosis and leave behind scaffolding for new bone cells to invade, resulting in bony overgrowth.

Specifically, we studied the effects of noggin and chordin, two ligand-binding antagonists of BMP4. Chordin has been shown to effectively inhibit EBF by preventing chondrocytes from maturing and hypertrophying [18]. As BMP4 levels increase, noggin is produced in response to elevated BMP4 and is a highly regulated inhibitor that can delay EBF [19].

Methotrexate is the first-line therapy for the treatment of JIA. Early studies showed that methotrexate, a folic acid antagonist, which was originally developed as a chemotherapy, at lower doses can reduce inflammation and pain in arthritic joints [20, 21]. Despite this medication being the mainstay of treatment for disease, little is known about the mechanisms through which methotrexate delays disease progression. This prompted us to investigate the effects of methotrexate on the chondrocyte-like phenotype of JIA FLS. We aimed to establish that inhibition of BMP4 delays differentiation of FLS from persistent, ETB, and polyarticular JIA to a hypertrophic chondrocyte-like phenotype and that methotrexate can reduce BMP4 levels and prevent FLS from becoming hypertrophic chondrocyte-like cells. This is a novel role for methotrexate, as it can possibly inhibit FLS from undergoing EBF and contributing directly to joint growth disturbances seen in affected joints.

## Materials and methods

### Selection of samples

Synovial fluid samples were obtained from our Institutional Review Board-approved repository. Patients who underwent clinically indicated arthrocentesis were offered inclusion into the repository, and informed consent was obtained. We selected primary cells from three subjects with persistent oligoarticular JIA, three subjects who eventually extended to a polyarticular disease course (termed extended-to-be), and three subjects who had polyarticular arthritis at time of diagnosis. All samples had no prior steroid injections and were on nonsteroidal anti-inflammatory drugs (NSAIDs) or no medications at time synovial fluid was obtained.

### Cell culture

Synovial fluid was spun at 500 rpm for 5 minutes and cell pellets were plated in T-25 culture flasks with 15% fetal bovine serum/Dulbecco’s modified Eagle’s medium. Initial primary cultures were passaged into T-75 flasks and, at confluence, passaged 3 more times, to ensure FLS are the predominant cell type in culture [22]. FLS from persistent oligoarticular, extended-to-be oligoarticular, and polyarticular JIA were grown in medium containing 15% FBS. At sub-confluence, media was removed, and cells were incubated overnight in growth medium containing 0.2% serum. The serum-starved cells were then seeded on 6-well plates for experiments. Cells were stimulated with either 100 μM methotrexate, 1000 ng/ml of recombinant human noggin protein (R&D Systems 6057-NG), 1000 ng/ml of recombinant human chordin protein (R&D Systems 1808-NR), or normal media for 24 hours. After 24 hours, cell media supernatants were obtained from each condition. Concentrations of methotrexate were determined based on previously published work in which FLS isolated from RA patients were treated with methotrexate [23, 24]. Concentrations of noggin and chordin were determined by our previously published work [16, 25].

### Enzyme-linked immunosorbent assay (ELISA)

Protein concentrations from cell culture supernatants were measured using Bradford assay. All ELISAs were performed according to manufacturer’s protocols for sample dilution and procedures. ELISA kits: collagen II (LifeSpan Biosciences, Inc. LS-F26824), collagen X (LifeSpan Biosciences, Inc. LS-F13131), aggrecan (RayBiotech, Inc. ELH-ACAN), BMP4 (R&D Systems, Inc. DBP-400). A power analysis was performed, and the results are included as supplemental material (Supplemental Table 1). Three biological FLS replicates were used for each JIA subtype for a total of nine samples in each condition. Briefly, cell culture supernatant samples from persistent, ETB, and polyarticular we plated in triplicate. Optical density readings were converted to ng/ml using standard curves. Averages and standard deviations were calculated for each sample using Excel. Averages and standard deviations for each JIA subtypes were calculated using Excel. Protein concentration measurements were plotted after 24 h in culture, and t-tests were used to determine statistical differences between two groups. Specifically, stasticial comparisons were only performed between either two subtypes of JIA or two conditions for each JIA subtype. For detailed methods please refer to our previously published work [16, 25].

## Results

### FLS from JIA subtypes express chondrocyte markers and polyarticular FLS are most like hypertrophic chondrocytes as evidenced by increased collagen X expression

**Fig. 2:**
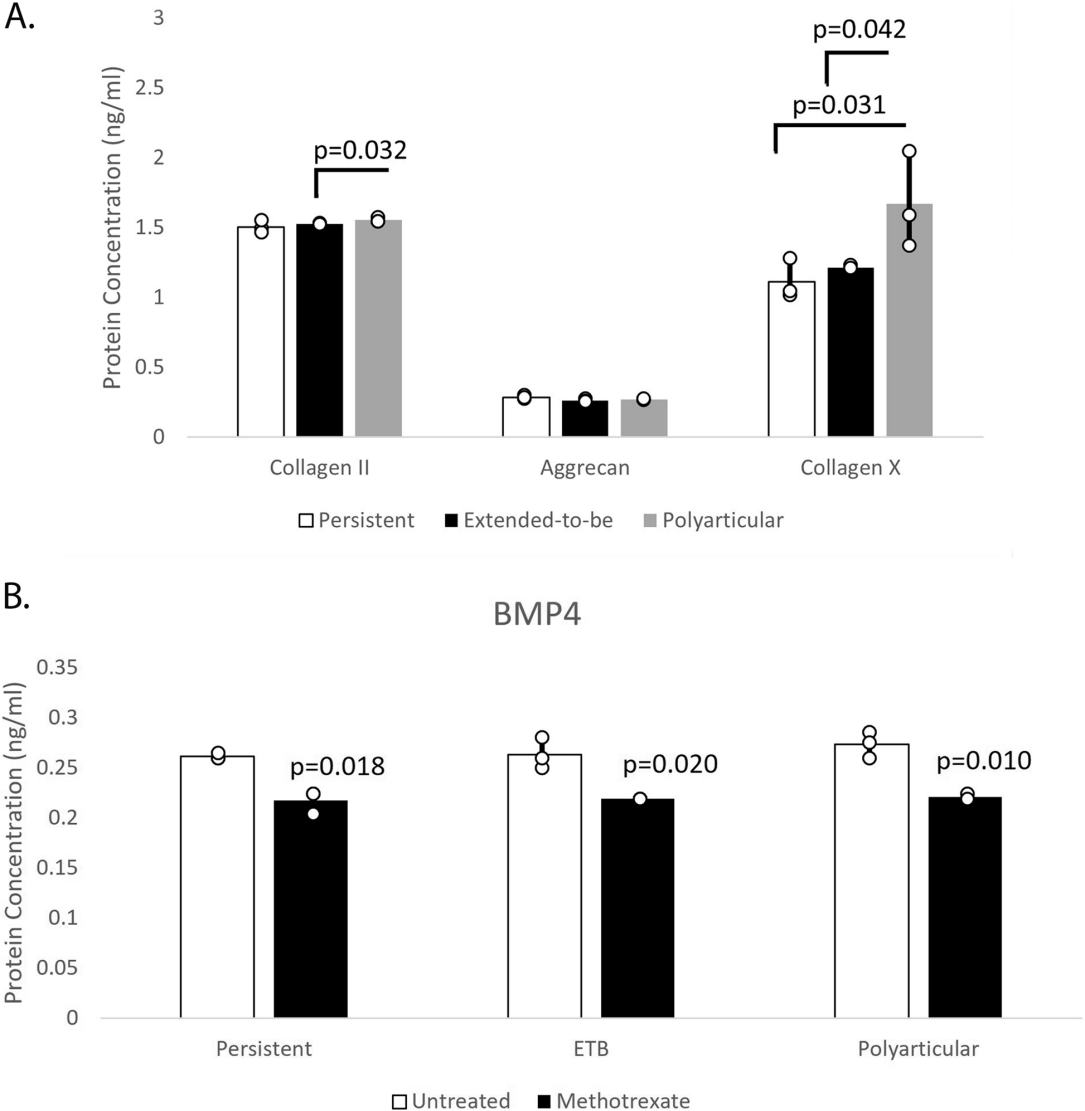
Chondrocyte marker expression in persistent, ETB, and polyarticular samples and methotrexate treatment on FLS from persistent, ETB, and polyarticular. Col2 was significantly increased in polyarticular JIA FLS when compared to ETB JIA FLS (FC = 1.02; *p* = 0.032) (**A**). ColX was significantly increased in polyarticular JIA FLS when compared to both persistent JIA FLS (FC = 1.50; *p* = 0.031) and ETB JIA FLS (FC = 1.37; *p* = 0.042) (**A**). In persistent JIA FLS, BMP4 protein expression decreased by − 1.20-fold in treated cells compared to untreated cells (*p* = 0.018) (**B**). BMP4 decreased by − 1.20-fold in ETB JIA FLS treated cells compared to untreated cells (*p* = 0.020) (**B**). Lastly, in the polyarticular JIA FLS, cells treated with methotrexate had a − 1.24-fold decrease in total BMP4 protein compared to untreated cells (*p* = 0.010) (**B**)

We have previously shown that FLS from the persistent oligoarthritis subtype have a chondrocyte-like phenotype when compared to normal FLS and these chondrocyte-like characteristics could contribute directly to bony overgrowth seen in affected joint [15, 16]. Given these findings, we examined expression of proteins that signify the stages of chondrocyte differentiation. Specifically, we measured expression of collagen II (Col2), a marker of early chondrocytes, aggrecan (Acan), a marker of mature chondrocytes, and collagen X (ColX), a marker of hypertrophic chondrocytes. Using ELISA, we determined protein expression secreted by FLS from the persistent, ETB, and polyarticular JIA FLS. Col2 was significantly increased in polyarticular JIA FLS when compared to ETB JIA FLS (FC = 1.02; *p* = 0.032) (Fig. 2A). ColX was significantly increased in polyarticular JIA FLS when compared to both persistent JIA FLS (FC = 1.50; *p* = 0.031) and ETB JIA FLS (FC = 1.37; *p* = 0.042) (Fig. 2A). Polyarticular JIA FLS are most like hypertrophic chondrocytes compared to persistent and ETB JIA FLS suggesting that cells from the most severe disease course are highly transformed phenotypically.

### DMARD methotrexate is effective at lowering BMP4 protein levels in FLS across JIA subtypes

Given that BMP4 can drive EBF in hypertrophic chondrocytes [26] and that JIA FLS have a hypertrophic chondrocyte-like phenotype, we measured total BMP4 protein, using ELISA, in persistent, ETB, and polyarticular JIA FLS treated with methotrexate. Methotrexate treated cells had significantly less BMP4 protein compared to untreated cells (Fig. 2B). Specifically, in persistent JIA FLS, BMP4 protein expression decreased by − 1.20-fold in treated cells compared to untreated cells (*p* = 0.018). BMP4 decreased by − 1.20-fold in ETB JIA FLS treated cells compared to untreated cells (*p* = 0.020). Lastly, in the polyarticular JIA FLS, cells treated with methotrexate had a − 1.24-fold decrease in total BMP4 protein compared to untreated cells (*p* = 0.010). Methotrexate inhibits BMP4 expression in JIA FLS.

### DMARD methotrexate is effective at abrogating the hypertrophic chondrocyte-like phenotype observed in FLS across JIA subtypes

**Fig. 3:**
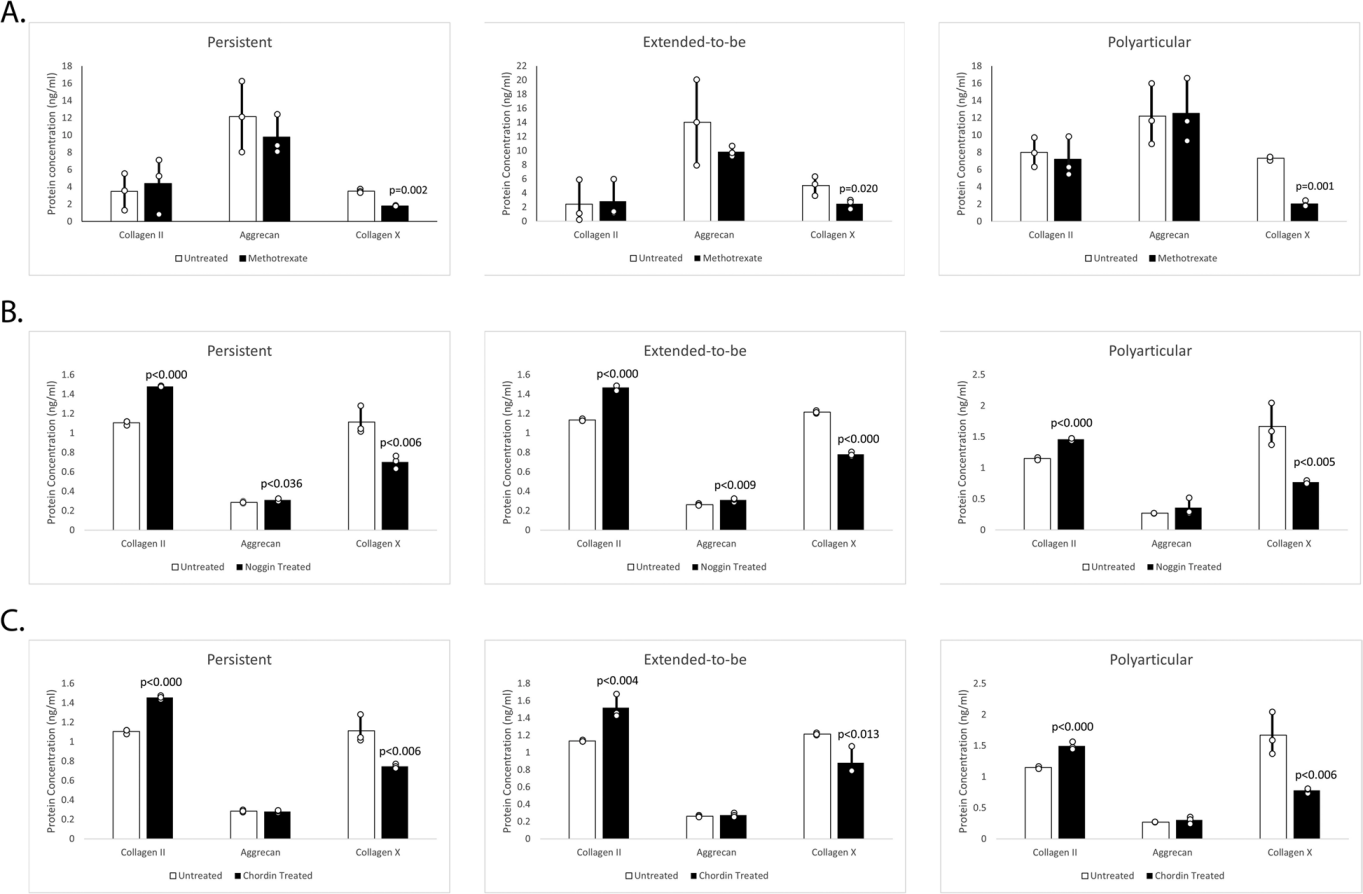
Chondrocyte marker expression after treatment with methotrexate and BMP4 inhibitors. In persistent JIA FLS, there was a − 1.97-fold decrease in ColX expression in cells treated with methotrexate compared to untreated (*p* = 0.002) (**A**). Methotrexate reduced ColX expression in ETB JIA FLS by − 2.03-fold when compared to untreated cells (*p* = 0.020) (**A**). In polyarticular JIA FLS, cells treated with methotrexate had decreased expression of ColX by − 3.63-fold compared to untreated cells (**A**). In persistent JIA FLS, Col2 (FC = 1.34; *p* < 0.000) and Acan (FC = 1.09; *p* = 0.036) protein concentration significantly increased in cells treated with noggin compared to untreated cells (**B**). ColX (FC = -1.58; *p* = 0.006) protein expression was significantly decreased in cells treated with noggin compared to untreated cells (**B**). In ETB JIA FLS, Col2 (FC = 1.30; *p* < 0.000) and Acan (FC = 1.18; *p* = 0.009) protein expression was significantly increased in cells treated with noggin compared to untreated cells (**B**). ColX (FC = -1.56; *p* < 0.000) protein concentration significantly decreased in cells treated with noggin compared to untreated cells (**B**). In polyarticular JIA FLS, Col2 (FC = 1.27; *p* < 0.000) protein concentration significantly increased in cells treated with noggin compared to untreated cells. ColX (FC = -2.16; *p* = 0.005) protein expression was significantly decreased in cells treated with noggin compared to untreated cells (**B**). In persistent JIA FLS, Col2 (FC = 1.32; *p* < 0.000) was significantly elevated in cells treated with chordin compared to untreated cells while ColX (FC = -1.45; *p* = 0.006) protein levels significantly decreased in cells treated with chordin compared to untreated cells (**C**). In ETB JIA FLS, Col2 (FC = 1.34; *p* = 0.004) was significantly elevated in cells treated with chordin compared to untreated cells while ColX (FC = -1.38 *p* = 0.013) protein levels significantly decreased in cells treated with chordin compared to untreated cells (**C**). In ETB JIA FLS, Col2 (FC = 1.30; *p* = 0.004) was significantly elevated in cells treated with chordin compared to untreated cells while ColX (FC = -2.14 *p* = 0.006) protein levels significantly decreased in cells treated with chordin compared to untreated cells (**C**)

Since methotrexate significantly reduced BMP4 expression in FLS from all three JIA subtypes, we examined its effect on chondrocyte markers using ELISA. Specifically, we measured the concentration of Col2, Acan, and ColX in FLS from each subtype. While there were no significant differences in Col2 or Acan expression in methotrexate treated cells from any of the subtypes, methotrexate did significantly lower expression of ColX, the hypertrophic chondrocyte marker, in FLS from persistent, ETB, and polyarticular samples when compared to respective untreated cells (Fig. 3A). In persistent JIA FLS, there was a − 1.97-fold decrease in ColX expression in cells treated with methotrexate compared to untreated (*p* = 0.002). Methotrexate reduced ColX expression in ETB JIA FLS by − 2.03-fold when compared to untreated cells (*p* = 0.020). Lastly, in polyarticular JIA FLS, cells treated with methotrexate had decreased expression of ColX by − 3.63-fold compared to untreated cells (*p* = 0.001).

### BMP4 ligand inhibitors noggin and chordin effectively reverse the hypertrophic chondrocyte-like phenotype in FLS from each JIA subtype

To demonstrate that the inhibition of BMP4 is necessary to cause JIA FLS to dedifferentiate away from a hypertrophic chondrocyte-like phenotype, using ELISA, we measured chondrocyte marker expression in JIA FLS treated with either noggin or chordin, two prominent BMP4 inhibitors that act by binding directly to BMP4 ligand. In persistent JIA FLS, Col2 (FC = 1.34; *p* < 0.000) and Acan (FC = 1.09; *p* = 0.036) protein concentration was significantly increased in cells treated with noggin compared to untreated cells (Fig. 3B). ColX (FC = -1.58; *p* = 0.006) protein expression was significantly decreased in cells treated with noggin compared to untreated cells (Fig. 3B). Similarly, in persistent JIA FLS, Col2 (FC = 1.32; *p* < 0.000) was significantly elevated in cells treated with chordin compared to untreated cells while ColX (FC = -1.45; *p* = 0.006) protein levels significantly decreased in cells treated with chordin compared to untreated cells (Fig. 3C).

In ETB JIA FLS, Col2 (FC = 1.30; *p* < 0.000) and Acan (FC = 1.18; *p* = 0.009) protein concentration significantly increased in cells treated with noggin compared to untreated cells (Fig. 3B). ColX (FC = -1.56; *p* < 0.000) protein expression was significantly decreased in cells treated with noggin compared to untreated cells. Similarly, in ETB JIA FLS, Col2 (FC = 1.34; *p* = 0.004) was significantly elevated in cells treated with chordin compared to untreated cells while ColX (FC = -1.38 *p* = 0.013) protein levels significantly decreased in cells treated with chordin compared to untreated cells (Fig. 3C).

In polyarticular JIA FLS, Col2 (FC = 1.27; *p* < 0.000) protein concentration significantly increased in cells treated with noggin compared to untreated cells (Fig. 3B). ColX (FC = -2.16; *p* = 0.005) protein expression was significantly decreased in cells treated with noggin compared to untreated cells. Similarly, in polyarticular FLS, Col2 (FC = 1.30; *p* = 0.000) was significantly elevated in cells treated with chordin compared to untreated cells while ColX (FC = -2.14 *p* = 0.006) protein levels significantly decreased in cells treated with chordin compared to untreated cells (Fig. 3C). Regardless of subtype, BMP4 ligand inhibitors, noggin and chordin, effectively cause JIA FLS to dedifferentiate away from a hypertrophic chondrocyte-like phenotype as noted by decreased expression of ColX and increased expression of Col2.

## Discussion

In this study, we show that methotrexate simulates the processes of well-characterized BMP4 inhibitors and that inhibition of BMP4 by methotrexate can reverse the hypertrophic chondrocyte-like phenotype of JIA FLS. Previously, we have shown that FLS from the persistent oligoarthritis disease course overexpress BMP4 and have a chondrocyte-like phenotype [15, 16, 25] when compared to normal FLS; therefore, we investigated the chondrocyte-like phenotype in three different JIA subtypes: persistent, ETB, and polyarticular. As disease course progresses, FLS express more collagen X. We hypothesize that JIA FLS are capable of contributing directly to bony overgrowth seen in affected joints of patients with JIA and that this occurs through endochondral bone formation, a process in which hypertrophic chondrocytes undergo apoptosis and provide the scaffolding for new bone to invade [27]. To our knowledge, this is the first study to isolate FLS from different JIA subtypes and propose a possible mechanism through which methotrexate can inhibit BMP4 protein expression.

While methotrexate has been used as a first-line therapy in the treatment of both Rheumatoid Arthritis (RA) and JIA since the late 1980s, little is known about the mechanisms through which this medication works in the treatment of these diseases. Recently, studies have shown that methotrexate suppresses JAK/STAT signaling, and prevents phosphorylation of STAT proteins, proposing a possible mechanism for how methotrexate reduces inflammation in RA and JIA [28]. Furthermore, STAT3, a member of the JAK/STAT pathway has been well characterized as having a critical role in bone homeostasis [29, 30]. In this study, we show that methotrexate can effectively decrease BMP4, a member of the TGFβ superfamily, protein expression in FLS from persistent, ETB, and polyarticular JIA. Elliot et al. measured BMP4 gene expression levels on human bone cells treated with methotrexate using RT-PCR. Human bone cells treated with methotrexate after mechanical stimulation had significantly reduced mRNA expression of BMP4 after 24 hours [31]. This is the first study to examine the effect of methotrexate on FLS from patients with JIA. We showed that hypertrophic chondrocyte marker, ColX was also significantly reduced in FLS from persistent, ETB, and polyarticular JIA when treated with methotrexate.

To strengthen the evidence that methotrexate acts as a BMP4 inhibitor, we demonstrated the traditional mechanisms of BMP4 antagonists and their effectiveness at lowering BMP4 expression in JIA FLS and reverting JIA FLS away from a hypertrophic chondrocyte-like phenotype. BMP4 is effectively inhibited by either noggin or chordin and lower BMP4 levels prevent JIA FLS from expressing ColX.

## Conclusion

Data from this study suggests that methotrexate, when administered to JIA FLS in culture, can mimic the behavior of a BMP4 inhibitor and consequently preclude these cells from contributing to joint growth disturbances associated with JIA. Our data also suggests that targeting BMP4 inhibition can cause FLS from persistent, ETB, and polyarticular JIA to dedifferentiate away from a hypertrophic chondrocyte-like phenotype. While we established a relationship between methotrexate and BMP4, we recognize that this study has limitations. The FLS were isolated from synovial fluid from patients with JIA and were subject to culture conditions; however, we tried to limit this by serum starving cells prior to administration of inhibitors or methotrexate. Although we examined cells isolated in a cell culture environment, we believe this data reveals a possible novel mechanism through which methotrexate can inhibit BMP4, a key signaling protein that is needed for endochondral bone formation.

### Supplementary Information

**Additional file 1: Table S1.** Power analysis on ELISA data to determine sample number. Power of 0.80 with a 0.05% error rate is reached based on sample number presented under each column for each JIA subtype where *n* = number of samples needed to reach power for a particular treatment.

## Data Availability

All data pertaining to the manuscript is discussed within manuscript; however, we can make data available upon request.

## Abbreviations

JIA: Juvenile idiopathic arthritis
FLS: Fibroblast-like synoviocytes
BMP4: Bone morphogenetic protein 4
EBF: Endochondral bone formation
ETB: Extended-to-be
Col2: Collagen II
ColX: Collagen X
Acan: Aggrecan
