# Supplementary figures and images for: Methotrexate inhibits BMP4 and abrogates the hypertrophic chondrocyte phenotype of synovial fibroblasts in juvenile idiopathic arthritis
Source: Pediatr Rheumatol Online J. 2024 Jan 2;22:6. doi: 10.1186/s12969-023-00940-6 (PMC10763212; doi:10.1186/s12969-023-00940-6)

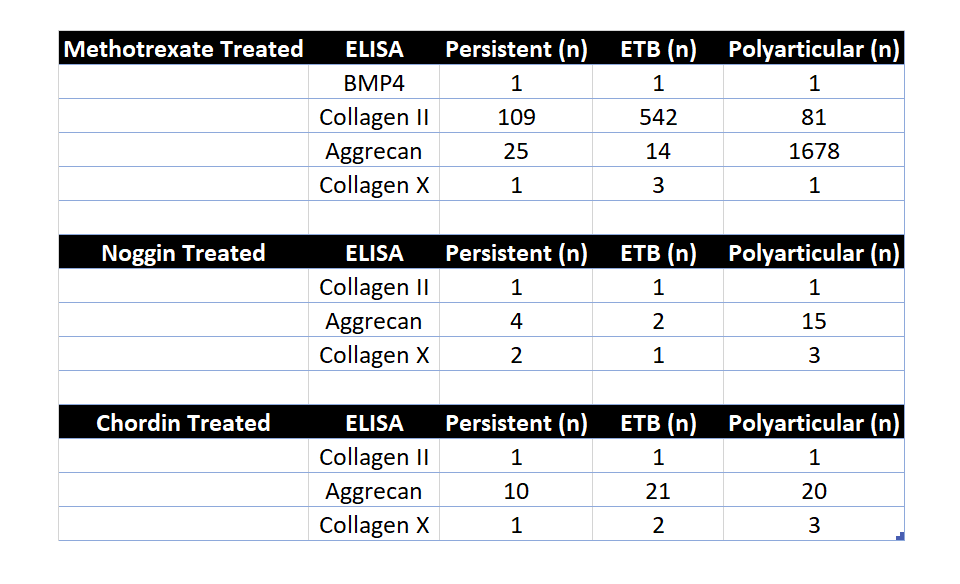

Supplement: Supplementary file 1 — Additional file 1: Table S1. Power analysis on ELISA data to determine sample number. Power of 0.80 with a 0.05% error rate is reached based on sample number presented under each column for each JIA subtype where n = number of samples needed to reach power for a particular treatment. [file 12969_2023_940_MOESM1_ESM.tif]
